# Rabies postexposure prophylaxis survey--Kentucky, 1994.

**DOI:** 10.3201/eid0302.970216

**Published:** 1997

**Authors:** M Auslander, C Kaelin

**Affiliations:** Kentucky Department for Public Health, Frankfort, USA.

## Abstract

A survey of rabies postexposure prophylaxis administered by local health departments for a 1-year period showed that very few patients received treatment as a result of exposure to a confirmed rabid animal. Most prophylaxis was administered for contact with domestic animals in situations where existing recommendations for quarantine or laboratory testing of the animal were not followed. Because rabies in domestic animals in Kentucky is uncommon, these findings suggest that had the existing recommendations been followed, the prophylaxis would have been unnecessary in most cases.


### Rabies Postexposure Prophylaxis Survey—Kentucky, 1994

A survey of rabies postexposure prophylaxis administered by local health departments for a 1-year period showed that very few patients received treatment as a result of exposure to a confirmed rabid animal. Most prophylaxis was administered for contact with domestic animals in situations where existing recommendations for quarantine or laboratory testing of the animal were not followed. Because rabies in domestic animals in Kentucky is uncommon, these findings suggest that had the existing recommendations been followed, the prophylaxis would have been unnecessary in most cases.

Rabies postexposure prophylaxis (PEP) is expensive, not totally free of risk, and overused (1). A national public health objective for the year 2000 is to reduce the number of prophylaxis treatments by 50% (2). In Kentucky, where PEP is administered in public and private settings, there are no baseline data on PEP use.

A survey of local health departments was used to determine the nature of each patient's exposure to rabies. The number of PEP treatments administered by all providers in Kentucky was estimated from local health department information on rabies biologics purchases and use.

### Survey and Sales Summary

In May 1995, the 1994 invoices of the Kentucky Department for Health Services, Vaccine Depot, were reviewed to determine which local health departments received 1.0 ml doses of human diploid cell vaccine for PEP. (Local health departments used 1.0 ml human diploid cell vaccine for PEP only, and 0.1 ml human diploid cell vaccine intradermally for all rabies preexposure prophylaxis). Data from two large health departments that acquired their vaccine directly from the manufacturer rather than from the Vaccine Depot were included in the survey. In June 1995, local health departments that had administered at least one PEP during 1994 were asked to review the records of patients receiving PEP. Information (patient's age and sex, the number of doses of human diploid cell vaccine, whether human rabies immune globulin was administered, exposure information, and method of payment for the treatment) collected on each patient was recorded on a standardized form by the same telephone surveyor during a follow-up telephone call. All data were entered into an Epi Info Version 5.0 record file and analyzed in either the Analysis or Statcalc Programs for summary statistics and/or odds ratios, confidence intervals, Fisher's exact test, or Chi-square at the .05 significance level (3).

A sales record summary for human diploid cell vaccine sold to all providers in Kentucky was obtained from the only manufacturer of human rabies vaccine recording any sales in Kentucky that year (Connaught Laboratories, Inc., Swiftwater, PA). The number of PEPs administered in the state by all providers was estimated by comparing local health department purchases and use with the total number of human diploid cell vaccine 1.0 ml doses sold to other providers with Kentucky addresses.

### PEP Administration Profile

Vaccine Depot records indicated that 28 health departments treated a total of 97 patients. The number of PEP regimens administered per health department ranged from 1 to 23 with a median of 1 PEP for the year. Fifty-two (53.6%) of the patients were male (Table 1); the median age was 28 years (range 2 to 71); 34 (35.1%) patients were younger than 18 years of age; 59 (60.8%) were older than 18 years of age; and for 4 (4.1%), age was unknown. No significant differences were observed in the type of animal exposure by sex or age. Seven patients (7.2%) had previously received PEP and were treated with two to three doses of human diploid cell vaccine and no human rabies immune globulin.

Urban health departments (in the three metropolitan statistical areas of the state) were more likely to administer PEP than rural health departments (odds ratio = 1.54, confidence interval = 1.01, 2.33) (4). Patients did not significantly

Table 1. Characteristics of local health department patients receiving rabies postexposure prophylaxis

| | | |
|---|---|---|
| Sex | | |
| | Male | 52 |
| | Female | 43 |
| | Unspecified | 2 |
| Age[a] | Youth (2 - 10) | 19 |
| | Adolescent (11 - 17) | 15 |
| | Adult (18-71) | 59 |
| | Unspecified | 4 |
| Health department location[b] | | |
| | Urban | 48 |
| | Rural | 49 |
| Previously immunized | | 6 |
| Animal exposure | | |
| | Wild | 15 |
| | Domestic (50 dogs, 29 cats, 1 horse) | 80 |
| | Unspecified | 2 |
| Type of exposure | | |
| | Bite or contact with saliva | 72 |
| | No contact with saliva | 17 |
| | Unspecified | 8 |
| Treatment payer | | |
| | Private insurance | 39 |
| | Medicaid | 7 |
| | Medicare | 3 |
| | Patient | 14 |
| | Other (employer, worker's compensation) | 3 |
| | Unspecified | 6 |
| | No reimbursement | 25 |

(N=97)

[a] $\bar{x}$ = 28 yrs.

[b] Health departments in urban areas, as defined by the 1990 census of population for Kentucky. Metropolitan statistical areas were more likely to administer PEP than rural departments. (p=.033)

differ in age, sex, or type of exposure between urban and rural health departments.

For 25 (25.8%) of the patients, local health department funds covered the expense of PEP treatments; no payment was received from private insurance, Medicaid, Medicare, or the patient. There were no significant differences in payment characteristics between urban and rural health department patients.

Bite exposures were responsible for 71 (73.2%) of the 97 PEP treatments, 18 (18.6%) exposures were scratches, licks, or "other," and 8 (8.2%) exposure types were not recorded. Domestic animals accounted for 80 (82.5%) of the exposures treated.

### Type of Animal Exposure

Sixty-four (77.1%) of 83 animals involved in these incidents were not available for observation or testing. For wild animals, testing was performed in 3 (20%) of 15 incidents. Testing or observation occurred in only 16 (20.0%) of 80 domestic animal exposures.

Stray domestic animals accounted for 26 (26.8%) of all exposures. Another 19 (19.6%) of the incidents involved owned dogs that were unavailable for testing or observation. Unavailability for testing was due to severe brain damage caused by clubbing or gunshot by irate owners, death and disposal of the animal without testing, or the animal's escape. For 36 (37%) incidents, the reason for not testing or observing the animal was not specified.

Thirteen (13.4%) of the patients were exposed to an animal that was tested and found to be positive for rabies, and two of these patients had bite exposures. The remaining exposures to these rabies-positive animals were either low-risk exposures or not true exposures (Table 2).

Table 2. Patients receiving postexposure prophylaxis for exposure to a confirmed rabid animal in Kentucky, 1994

| Species | Type of exposure | Previous history of prophylaxis |
|---|---|---|
| Bat | Bite | No |
| Cat[a] | Mucus & Saliva | Yes[b] |
| Cat[a] | Mucus & Saliva | No |
| Cat[a] | Cleaned exam table | No |
| Cat[a] | Cleaned exam instruments | No |
| Dog[c] | Bite | Yes |
| Dog[c] | Touch | Yes |
| Dog[c] | Touch | Yes |
| Dog[c] | Touch | Yes |
| Dog[c] | Touch | No |
| Dog[c] | Touch | No |
| Horse | Sutured wound | Yes[b] |
| Skunk | Touch | No |

[a] Same cat

[b] Veterinarian with history of preexposure prophylaxis

[c] Same dog

### Total Estimate of State Rabies Postexposure Prophylaxis

Kentucky sales in 1994 for human diploid cell vaccine 1.0 ml to nonmilitary providers and distributors totaled 1,603 doses. The health departments ordered 700 of these doses, of which

445 were used for PEP in that same year. The other doses remained as inventory. Assuming that other users administered human diploid cell vaccine 1.0 ml in a similar proportion (445/700 = 0.64), the private sector administered 578 doses (903 x .64) of human diploid cell vaccine 1.0 ml. Comparing actual local health department use of human diploid cell vaccine 1.0 ml and estimated use by others, local health departments administered 43.5% (445/([445+578]) of the human diploid cell vaccine 1.0 ml used in the state in 1994. Therefore, the estimated total number of PEP patients in the state is 223 (97/.435) for 1994.

Exact total costs for PEP administration cannot be calculated since most treatments were made by private providers. The actual cost of biologics to local health department patients in 1994 was $68,850. Estimated costs of biologics used by private providers (based on estimates of hospital pharmacy costs in Connecticut in 1994) would be $180,180 for a typical patient (126 patients x $1,430) (5). Estimated total costs of biologics is $249,030. Unknown costs include medical and hospital care, local health department investigation of the incident, state health department consultations, and loss of work income by the patient.

### Study Limitations

Because records at the local health departments were not always complete or as detailed as desired, certain variables could not be analyzed for all 97 cases; information about why the suspect animal was not tested or observed for rabies was absent from more than 10% of the cases. Since no detailed information was obtained from the private sector, we assumed that the number of doses used per patient, inventory, waste, spoilage, and other factors influencing PEP use in the private sector were similar to those in the public sector. Kentucky residents receiving PEP in another state and out-of-state residents receiving PEP in Kentucky would not be specifically accounted for in our estimate.

The difference in urban versus rural PEP administration could be due to differences in the number of animals or bite incidents; however, the number of animals or animal bites statewide is not known. An investigation of prescribing practices of full-time physicians at large, urban health departments and part-time or contract physicians at small, rural health departments might determine if these practices contributed to treatment disparity.

### Guidelines and Noncompliance

Guidelines for determining exposures that warrant PEP exist (6,7). Ideally, any animal involved in a human exposure should be confined and observed or tested for rabies, whichever is appropriate. It is understandable that most of the wild animals might have escaped and not be available for testing. However, the large proportion of domestic animals unavailable for testing indicates inappropriate handling of the incident or a breach of existing laws (5-7).

Six people received PEP due to exposure to a single dog with laboratory-confirmed rabies. This particular incident illustrates how “anything that can go wrong will go wrong.” First, the dog had been vaccinated by the owner. It is illegal for individual owners to vaccinate their own dogs in Kentucky (8). Second, the vaccine may have failed for any number of reasons, including vaccine failure, improper handling/administration of the vaccine, or failure to vaccinate. Third, only one of these patients was bitten; the other five reported only touching the dog and probably were not exposed. Fourth, none of these patients had insurance or was able to pay for treatment; thus the local health departments spent several thousand dollars in unbudgeted expenses. Furthermore, four of these patients had received PEP before.

Noncompliance with existing public health recommendations and laws contributes to the number of rabies exposure incidents in Kentucky. PEP administration in Kentucky could be reduced if existing recommendations and laws were adhered to by the public and health care providers. Accurate and complete record keeping is essential for assessing the use of PEP. Additionally, making PEP a notifiable (reportable) condition would allow public health agencies to assess PEP administration in the private sector.

**Michael Auslander and Colleen Kaelin**
Kentucky Department for Public Health,
Frankfort, Kentucky, USA

### References

1. Noah D, Smith M, Gotthardt J, Krebs J, Green D, Childs J, et al. Mass human exposure to rabies in New Hampshire: exposures, treatment, and cost. Am J Public Health 1996;86:1149-51.
2. Healthy People 2000: National Health Promotion and Disease Prevention Objectives. Washington (DC): Public Health Service; 1990:122. DHHS publication PHS 91-50213.
3. Dean A, Dean J, Burton A, Dicker R. Epi Info, Version 5: a word processing, database, and statistics program for epidemiology on microcomputers. Stone Mountain (GA): USD, Inc., 1990.
4. Census of Population and Housing, 1990. Summary Tape File 3, Prepared by the Data Services Division, Bureau of the Census. Washington (DC): U.S. Bureau of the Census; 1990.
5. Centers for Disease Control. Rabies postexposure prophylaxis - Connecticut, 1990-1994. MMWR Morb Mortal Wkly Rep 1996;45:232-34.
6. Centers for Disease Control. Rabies prevention - United States, 1991. MMWR Morb Mortal Wkly Rep 1991;40:1-19.
7. Centers for Disease Control and Prevention. Compendium of Animal Rabies Control, 1996. MMWR Morb Mortal Wkly Rep 1996;45:1-9.
8. Kentucky Revised Statutes. Rabies Control. KRS 258.005-.090; 1994.
